# Aneurysmal subarachnoid hemorrhage complicating spinal subarachnoid hematoma causing acute cauda equina syndrome: a case report

**DOI:** 10.1186/s12883-023-03404-2

**Published:** 2024-01-02

**Authors:** Heng Ni, Yu Zheng, Shanshan Lu, Zhenyu Jia, Haibin Shi, Sheng Liu, Linbo Zhao

**Affiliations:** 1 Department of Interventional Radiology, The First Affiliated Hospital of Nanjing Medical University, 300 Guangzhou Rd, 210029 Nanjing, China; 2 Department of Radiology, The Affiliated YiXing Hospital of Jiangsu University, 75 Tongzhenguan Rd, 214200 Wuxi, China; 3 Department of Radiology, The First Affiliated Hospital of Nanjing Medical University, 300 Guangzhou Rd, 210029 Nanjing, China; 4 Department of Interventional Radiology, The First Affiliated Hospital with Nanjing Medical University, 300 Guangzhou Road, 210029 Nanjing, Jiangsu Province China

**Keywords:** Aneurysmal, Subarachnoid hemorrhage, Spinal subarachnoid hematoma, Cauda equina syndrome

## Abstract

**Background:**

Spinal subarachnoid hematoma (SSH) is a known but rare entity that can cause cauda equina compression. The occurrence of SSH associated with aneurysmal subarachnoid hemorrhage has rarely been described in the literature.

**Case presentation:**

A 56-year-old woman presented with subarachnoid hemorrhage secondary to a ruptured middle cerebral artery aneurysm and was managed with coiling embolization without stent assistance. There was no history of either lumbar puncture or the use of anticoagulants. The patient developed severe lumbago radiating to bilateral legs nine days after the procedure. Subsequent magnetic resonance imaging demonstrated a SSH extending from L5 to S2 and wrapping around the cauda equina. The patient was treated with intravenous methylprednisolone (250 mg/day) for four consecutive days, followed by a taper of oral prednisolone (20 mg/day) until complete recovery. Magnetic resonance imaging at one month follow-up revealed complete resolution of the SSH.

**Conclusions:**

Here, we report a case of acute cauda equina syndrome caused by a SSH after aneurysmal subarachnoid hemorrhage, which will facilitate timely intervention of patients with this disorder.

## Introduction

Spinal subarachnoid hematoma (SSH) is a rare entity causing spinal cord or nerve root compression [1]. In addition to idiopathic spontaneous development, SSH could be caused by trauma, iatrogenic procedures (most frequently due to lumbar puncture), arteriovenous malformations, arteriovenous fistula, spinal artery aneurysms, coarctation of the aorta, coagulopathies (as a result of pharmacotherapy or systemic diseases), neoplastic lesions, systemic lupus erythematosus, necrotizing vasculitis, or Behcet’s disease [2–10]. In most cases, the onset is acutely progressive and requires urgent surgical treatment, although some individuals with chronic SSH have been reported [11]. The comorbidity of SSH associated with intracranial aneurysmal subarachnoid hemorrhage (SAH) is extremely rare and the exact pathogenesis is unclear [12]. This paucity of clinical cases poses a challenge for its diagnosis and management in patients with cauda equina syndrome after aneurysmal SAH. This paucity of clinical cases poses a challenge for its diagnosis and management in patients with cauda equina syndrome after aneurysmal SAH. We present a case of acute cauda equina syndrome caused by a SSH after aneurysmal SAH. The clinical aspect, radiological images, pathogenesis, and management are described in this report.

## Case presentation

**Fig. 1:**
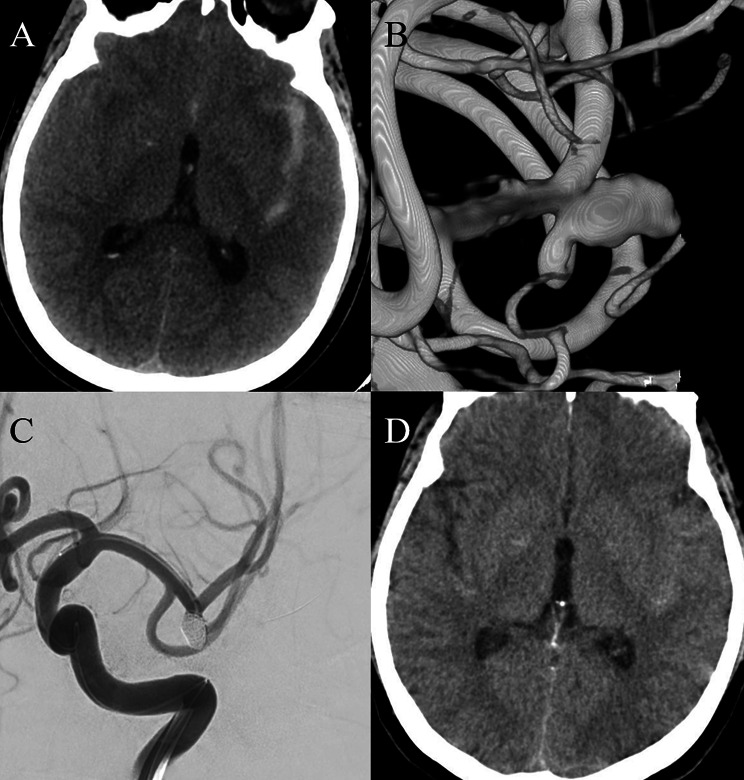
Computed tomography (**A**) and digital subtraction angiogram (**B**) revealed subarachnoid hemorrhage (SAH) secondary to a ruptured middle cerebral artery aneurysm. Patent was managed with successful endovascular coil embolization of the ruptured aneurysm without stent assistance (**C**). Computed tomography scan 7 days after onset of symptoms showed near-complete resolution of the SAH (**D**)

A 56-year-old woman with a history of hypertension suddenly developed a severe headache. She was taken to emergency room of our institution, where head computed tomography (CT) and CT angiography scans revealed SAH secondary to a ruptured middle cerebral artery (MCA) aneurysm (Fig. 1). The patient had a Hunt-Hess grade of 2 and a modified Fisher grade of 1 on admission. She then was managed with successful endovascular coil embolization of the ruptured left MCA aneurysm without stent assistance under general anesthesia. No lumbar puncture was performed during hospitalization. There was no evidence of delayed cerebral vasospasm. She did well neurologically and underwent a CT scan of the head 7 days after onset of symptoms, which showed complete resolution of the SAH.

**Fig. 2:**
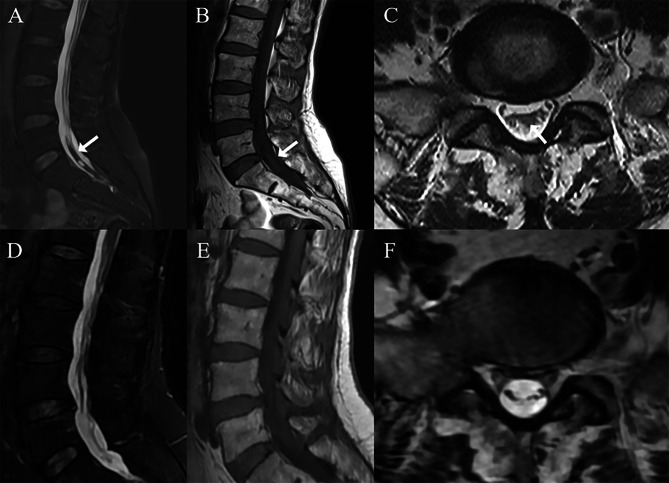
Magnetic resonance imaging (MRI) showed diffuse iso-signal intensity on T1-weighted images (**B**, arrow), and a low signal intensity on T2-weighted images (**A** + **C**, arrow) in the spinal canal at the level of L5 to S2, which suggested early subacute hemorrhage in the spinal subarachnoid space. Follow-up MRI (**D**-**F**) at one month revealed complete resolution of the hemorrhage

However, she presented with a severe low back pain radiating to bilateral legs nine days after the onset of SAH. Subsequent magnetic resonance imaging (MRI) showed diffuse iso-signal intensity on T1-weighted images, and a low signal intensity on T2-weighted images in the spinal canal at the level of L5 to S2, which suggested early subacute hemorrhage in the spinal subarachnoid space (Fig. 2). The spinal subarachnoid clot wrapped around the cauda equina with a loculated ventral cyst. There were no abnormal findings in the T2-weighted MRI images of the cervicothoracic spine. Laboratory examinations showed CRP 10.9 mg/L while other hematologic parameters.

in the routine blood and coagulation function tests were normal. A neurosurgical consultation was undertaken, and no surgical intervention was believed to be necessary. She was treated with intravenous methylprednisolone for four consecutive days (250 mg/day), followed by a taper of oral prednisolone (20 mg/day) until complete recovery. She was discharged with an improved clinical condition. At one month follow-up, she had a modified Rankin Scale (mRS) of 0. At one month post-discharge, MRI revealed complete resolution of the SSH.

## Discussion

Here, we present a case of acute cauda equina syndrome associated with SSH resulting from intracranial aneurysmal SAH treated by endovascular coil embolization. The pathogenesis of SSH is determined by the exact cause. In the cases of iatrogenic or traumatic SSH, some authors suggest that the major cause of hemorrhage is rupture of the radicular vessels, which may be responsible for the pressure variations within the spinal canal or lacerations by the spinal needle, especially in patients with defective coagulation [13, 14]. Generally, the cerebral spinal fluid (CSF) flow tends to dilute and wash away the blood and reduces the likelihood of clot formation in the spinal subarachnoid space; however, abundant bleeding or a diminished CSF may overwhelm this process [10]. Given an absence of predisposing procedures or other potential causes, the possible explanation for SSH in this case may be due to localized cerebrospinal fluid circulation disorders following intracranial SAH, leading to clot formation.

Clinical symptoms of SSH present as sudden back pain, acute sciatica, sensory disturbance, paraparesis, or sphincter disturbance, which are thought to be the results of external nerve root compression [11]. In addition, Kostov et al. proposed that abundant pooling of blood products in the lumbosacral subarachnoid space could irritate local nerve roots [12]. In this patient, the consequence after intracranial SAH may be due to the co-impact of compression and irritation of local nerve root and arachnoid from the clot in the spinal subarachnoid. MRI offers a great contribution in revealing the extent of bleeding and the degree of nerve root compression. In the cases with unknown origin, selective spinal angiography is essential to identify the underlying primary vascular diseases in the spinal canal [2].

Urgent decompressive surgery is recommended as the primary approach for SSH patients with progressive neurological deterioration, while conservative treatment is an acceptable option for selected patients with mild neurological impairment [10]. Komiyama et al. classified the location of the SSH as dorsal or ventral and they proposed that the ventral location of the hematoma, as observed in the present case, is less likely to cause severe spinal cord and nerve root compression [2]. However, the final decision to perform surgical decompression should depend on the patient’s neurological status, although several cases with spontaneous resolution of SSH were reported [15]. This case showed complete resolution of the hematoma during follow-up, with no observations of recurrence. Consistent with the results reported by Komiyama et al [2], patients are unlikely to experience further bleeding when the underlying cause and condition are well managed.

## Conclusion

Rapid subarachnoid bleeding from ruptured intracranial aneurysm may result in a formation of SSH, and these cases are easily neglected due to limited knowledge and mild clinical symptoms. Hence, this case helps to raise awareness that though the SSH is a rare comorbidity, a high degree of medical attention should be given to patients with acute sciatica symptoms following intracranial SAH.

## Data Availability

All data is available on reasonable request to the corresponding author.

## List of abbreviations

SSH: Spinal subarachnoid hematoma
SAH: Subarachnoid hemorrhage
CT: Computed tomography
MCA: Middle cerebral artery
mRS: modified Rankin Scale
CSF: Cerebral spinal fluid
